# Cardiomyocyte—Endothelial Cell Interactions in Cardiac Remodeling and Regeneration

**DOI:** 10.3389/fcvm.2018.00101

**Published:** 2018-07-26

**Authors:** Virpi Talman, Riikka Kivelä

**Affiliations:** ^1^Drug Research Program and Division of Pharmacology and Pharmacotherapy, Faculty of Pharmacy, University of Helsinki, Helsinki, Finland; ^2^Wihuri Research Institute and Translational Cancer Biology Program, Faculty of Medicine, University of Helsinki, Helsinki, Finland

**Keywords:** cardiomyocyte, endothelial cell, cell-cell interaction, cardiac regeneration, cardiac remodeling, cardiac cell therapy, cardiovascular gene therapy

## Abstract

The heart is a complex organ consisting of various cell types, each of which plays an important role in both physiological and pathophysiological conditions. The cells communicate with each other through direct cell-cell interactions and paracrine signaling, and both homotypic and heterotypic cell interactions contribute to the organized structure and proper function of the heart. Cardiomyocytes (CMs) and endothelial cells (ECs) are two of the most abundant cardiac cell types and they also play central roles in both cardiac remodeling and regeneration. The postnatal cell cycle withdrawal of CMs, which takes place within days or weeks after birth, represents the major barrier for regeneration in adult mammalian hearts, as adult CMs exhibit a very low proliferative capacity. Recent evidence highlights the importance of ECs not only as the most abundant cell type in the heart but also as key players in post-infarction remodeling and regeneration. In this MiniReview, we focus on blood vascular ECs and CMs and their roles and interactions in cardiac physiology and pathologies, with a special emphasis on cardiac regeneration. We summarize the known mediators of the bidirectional CM-EC interactions and discuss the related recent advances in the development of therapies aiming to promote heart repair and regeneration targeting these two cell types.

## The multicellular composition of the heart

The heart is a complex multicellular organ with specialized structures and cells to take care of their own “subprojects.” Its main function, to maintain the blood circulation, depends on its pump function, making cardiomyocytes (CMs) the central cardiac cell type in both normal and pathological conditions. Cardiomyocytes are generally divided into pacemaker cells and force-producing ventricular and atrial CMs. In order for the heart to function properly, additional cell types, such as blood and lymphatic endothelial cells (ECs), vascular smooth muscle cells (SMCs), fibroblasts, and pericytes are needed. Besides their structural role in the interior surfaces of blood vessels, vascular ECs are metabolically active, control vasomotor tone, and regulate angiogenesis (1, 2). Fibroblasts are connective tissue cells that produce constituents of extracellular matrix, while vascular SMCs and pericytes regulate blood flow in the cardiac vasculature. The proportions of cardiac cell types have been debated for decades, as technical limitations and the lack of cell-type specific markers have impeded accurate analysis. Traditionally, fibroblasts have been considered the most abundant cell population (3–5). Fairly recently, ECs were however reported to represent the major non-myocyte population (6), suggesting that their physiological and thereby therapeutic importance may be greater than previously appreciated. Of the ECs, about 95% are blood vascular and 5% lymphatic (6). There is recent evidence that lymphatic vessels also play an important role in the heart, especially after myocardial infarction (7, 8). However, no studies are available at the moment on the possible interplay between lymphatic ECs and CMs.

**Figure 1:**
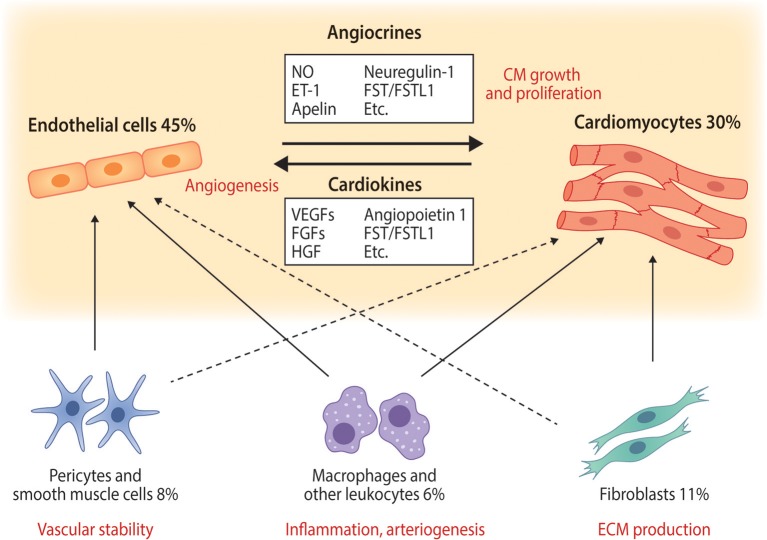
The main cardiac cell types and their interactions with endothelial cells and cardiomyocytes. Selected known paracrine factors mediating endothelial cell—cardiomyocyte cross-talk (angiocrines and cardiokines) are illustrated. The relative abundances of each cell type in normal adult mouse ventricular tissue are from (6) and represent those quantified in adult mouse heart. Solid arrows represent well known cell-cell interactions and dashed lines illustrate less well characterized but potentially important interactions. ET-1, endothelin-1; FGFs, fibroblast growth factors; FST, follistatin; FSTL1, follistatin-like 1; HGF, hepatocyte growth factor; NO, nitric oxide; VEGFs, vascular endothelial growth factors.

In the myocardium, CMs are physically connected and communicate with each other through gap junctions, adherens junctions, and desmosomes (9). However, they also communicate with other cell types in the heart through both direct physical contacts and paracrine signaling (Figure 1). Pathological conditions, such as hypertension or myocardial infarction (MI), elicit maladaptive responses in both CMs and non-myocytes, contributing to the deterioration of cardiac function. Cardiac fibrosis is one example of fibroblast-mediated phenomena playing a central role in the pathogenesis of heart failure (10). Heterotypic interactions between different cell types are known to cause or aggravate cardiac pathologies. Cell-cell interactions between fibroblasts and CMs that contribute to e.g., arrhythmogenesis have been extensively investigated and are reviewed in depth in (11–13). The interactions between the two most abundant cell types, ECs, and CMs, have however not been equally well characterized. In this MiniReview we focus on the importance and mechanisms of communication between blood vascular ECs and CMs as well as their therapeutic implications in cardiac repair and regeneration.

## Remodelling of cardiac vasculature and cardiomyocyte hypertrophy

Cardiac remodeling, i.e., remodeling of the left ventricular wall, can take place in physiological or pathological conditions (14). While physiological remodeling mainly includes CM hypertrophy, is reversible and improves cardiac function, pathological remodeling is accompanied with fibrosis and CM hypertrophy, atrophy, and apoptosis, and leads to deterioration of cardiac output. In physiological hypertrophy, the heart preserves its oxygen supply and balances the proportional increases in CM size and the extent of coronary microvasculature through angiogenesis. In heart failure, however, the pathological progression is associated with an imbalance between oxygen supply and demand, as CM hypertrophy is not matched by a corresponding increase in the vasculature (15). Identification of the mechanisms behind this mismatch between CM and EC growth could lead to discovery of new approaches to treat heart failure.

## Cardiac regeneration

Since the adult mammalian heart exhibits a very limited regenerative capacity (16, 17), a cardiac injury such as MI leads to fibrotic scarring and subsequent remodeling of the surrounding myocardium, eventually progressing to heart failure [see (10, 18)]. Some lower vertebrates, such as the zebrafish, as well as neonatal rodents can however regenerate their hearts (19, 20). Furthermore, it seems that the human heart also possesses this intrinsic regenerative capacity at the time of birth (21). The postnatal irreversible cell cycle arrest in mammalian CMs is connected to oxygen-rich postnatal environment and increased oxidative energy metabolism in cardiomyocytes, which promote oxidative stress and DNA damage (22). More detailed understanding of the mechanisms is however needed to unlock regenerative therapeutic possibilities [see (23–25)].

During cardiac regeneration in the zebrafish and neonatal mouse, new CMs are produced through proliferation of remaining CMs (20, 26, 27). Additionally, regeneration requires revascularization of the injured area, which occurs through proliferation and migration of vascular ECs from existing vessels in the surrounding myocardium (28). Remarkably, in zebrafish the ECs invade the injured area already in less than 1 day and rapid revascularization is indispensable for the induction of CM proliferation, suggesting that revascularization not only provides nutrients to the regenerating tissue, but also regulates the regenerative response. In liver and lung, ECs have been shown to be crucial for regeneration acting via paracrine secretion of angiocrines (29, 30). In line with this, both ECs and CMs in adult mouse hearts are unable to activate the injury-responsive neonatal gene regulatory networks, while MI-induced gene expression changes in fibroblasts and leukocytes are similar in neonatal and adult mouse hearts (31). This further highlights CMs and ECs as the main targets for regeneration-inducing therapies and calls upon detailed investigations clarifying their mutual interactions in the process of cardiac regeneration.

## Paracrine signalling between cardiomyocytes and endothelial cells

### Cardiokines affecting endothelial cells

Cardiomyocytes produce and secrete proteins and peptides for paracrine signaling with other cardiac cells and for endocrine signaling with peripheral tissues. It has been estimated that the heart secretes 30–60 different proteins or peptides, which are called cardiokines or cardiomyokines (32); however, the number may increase markedly as analysis techniques improve. Cardiokines include growth factors, endocrine hormones, cytokines, extracellular matrix proteins, and peptides, which are important for maintenance of normal heart growth and function and as signal mediators in response to various stresses. The well-known stress-induced cardiokines include natriuretic peptides A (ANP) and B (BNP), which act as cardioprotective factors mainly for CMs, but also exert effects on ECs (33).

The main cardiokine regulating EC activation and proliferation is vascular endothelial growth factor (VEGF) that binds to and activates the VEGF receptor 2 (VEGFR2, also known as KDR and FLK1) in ECs (34). VEGFR2 signaling induces angiogenesis, growth of new blood vessels from the pre-existing ones, improving circulation, and thereby oxygen and nutrient availability in deprived areas to match the need of e.g., growing or infarcted heart. Additional extensively studied cardiokines inducing cardiac angiogenesis include the other VEGF family members VEGF-B (35), VEGF-C (36), and placental growth factor (PlGF) (37), as well as fibroblast growth factors (FGFs) (38), hepatocyte growth factor (HGF) (39), and angiopoietin-1 (40). CMs also produce and secrete several members of the TGF-β superfamily, which elicit both cardioprotective and detrimental effects on the heart. Of these, follistatin like-1 is one of the best characterized, and it has been shown to affect both CMs and ECs [see (41)].

### Angiogenesis-induced cardiac hypertrophy

The first evidence indicating that angiogenesis induces cardiac hypertrophy came from a study by Tirziu et al. (42), where the authors showed that vascular growth without any other stimuli increases cardiac mass. Both VEGF-B and PlGF have been shown to induce cardiac angiogenesis accompanied by modest cardiac hypertrophy with preserved or improved function and, importantly, no progression into heart failure even in old transgenic animals (43–45). Currently, the molecular mechanisms mediating angiogenesis-induced CM hypertrophy are not known. Nitric oxide (NO) has been suggested to be important in this regulation, however, its blockade could not fully reverse the phenotype (44).

### Cardiac angiocrines mediating EC-CM crosstalk

It is estimated that humans have about 100,000 km of blood vessels, which most likely makes vasculature the largest “endocrine organ.” In response to physiological or pathological stimuli, ECs secrete a set of proteins and other factors, angiocrines, which act on the neighboring cell types. Recent data from other tissues provide strong evidence that ECs establish an instructive vascular niche, which stimulates organ growth and regeneration via paracrine signaling (29, 30, 46, 47). However, very little is known about the cardiac EC secretome (cardiac angiocrines) in response to physiological or pathological stimuli. While the concept of EC to CM signaling and its role in cardiac function has been extensively reviewed by Brutsaert already 15 years ago (48), we are still in the early phase of understanding the mechanisms of this crosstalk.

A growing number of EC-derived factors acting on CMs have been identified. One of the most studied over the years is NO, which is produced by many cell types in the heart and affects both vasculature and CMs. NO induces relaxation of vascular SMCs resulting in vasodilation (49), and can regulate contractile responses of CMs (50). Of the secreted proteins, neuregulin-1 (NRG-1), which belongs to the epidermal growth factor family and is expressed and released by cardiac microvascular ECs and perivascular cells, has received a lot of interest over the last 10 years. NRG-1 binds and activates ErbB receptors in CMs promoting cardioprotection and regeneration, and both NRG-1 and ErbB2 have been shown to promote CM proliferation and growth (51, 52). Apelin is another secreted peptide, which is highly EC-specific and expressed in sprouting vessels in the heart (53). It regulates cardiomyocyte hypertrophy and myocardial response to infarction through its receptor APJ (54, 55). Endothelin-1 (ET-1) was first identified as a potent vasoactive substance produced by porcine aortic ECs (56). It is produced by ECs, CMs, and fibroblasts, and its receptors are expressed in CMs, where it regulates CM contractility and maladaptive cardiac remodeling (57). There are also reports on other potential cardiac angiocrines such as prostacyclin, periostin, thrombospondins, follistatin/follistatin-like 1, angiotensin II, and connective tissue growth factor (58, 59), but their role and function in EC-CM crosstalk needs to be further validated with e.g., EC-specific gene deletion mouse models. In addition, pathological insults will lead to production of e.g., inflammatory and extracellular matrix proteins by several cell types in the heart, including ECs, but these are beyond the scope of this MiniReview.

### Physical cell-cell contacts and extracellular vesicles

In addition to paracrine mediators, also direct physical cell-cell contacts may play a role in EC-CM communication. Connexins are important in cell-cell communication and Cx43, Cx40, and Cx37 are expressed in both ECs and CMs [reviewed in (60)]. They form gap junctions between adjacent CMs enabling efficient electrical conduction. In ECs, connexins regulate e.g. leukocyte trafficking. Based on *in vitro* co-culture studies, connexins have also been suggested to mediate communication between ECs and CMs; however, more evidence is needed to prove the existence and relevance of direct EC-CM contacts *in vivo*. Furthermore, exosomes and other extracellular vesicles can carry non-coding RNAs such as microRNAs, long non-coding RNAs and circular RNAs, which have been shown to regulate cardiac remodeling and regeneration (61). Their roles and mechanisms in EC-CM communication have however not been characterized in detail.

## Therapeutic approaches

**Figure 2:**
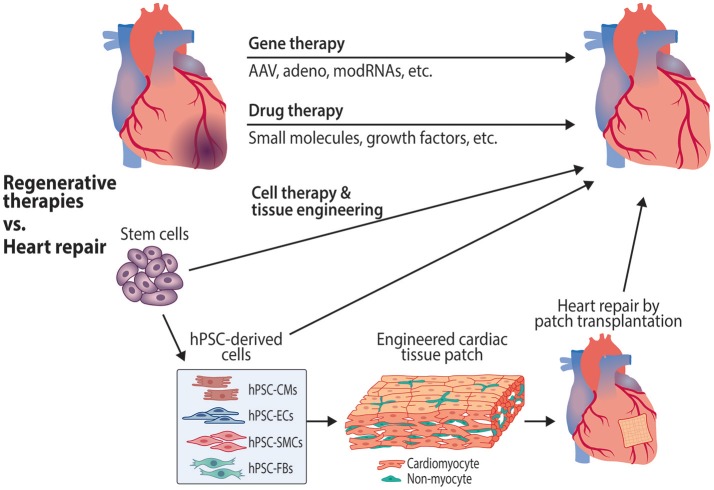
Therapeutic approaches targeting cardiomyocytes and endothelial cells to promote cardiac regeneration and repair. Gene therapies to induce revascularization and cardiac regeneration are being investigated using various approaches, including adeno-associated (AAV) and adenoviral vectors, as well as modified RNAs (modRNAs). Potential regenerative drug therapies that are in preclinical stages of drug discovery and development include small molecule compounds and biologics such as growth factors. Cell therapies include the use of stem cells, cardiovascular progenitor cells and stem cell-derived cardiovascular cells to repair the damaged myocardium. Tissue engineering approaches use stem cell-derived cardiomyocytes and other cell types to create organized tissue patches for transplantation. hPSC, human pluripotent stem cell; hPSC-CMs, hPSC-derived cardiomyocytes; hPSC-ECs, hPSC-derived endothelial cells; hPSC-SMCs, hPSC-derived smooth muscle cells; hPSC-FBs, hPSC-derived fibroblasts.

With the pressing and unmet need for effective treatments to reduce cardiovascular mortality, various approaches are being investigated as potential reparative and regeneration-inducing treatments. Therapeutic strategies aiming to induce vascular and cardiac regeneration include gene therapy and drug development, while cardiac repair is being pursued with cell therapy and tissue engineering approaches (Figure 2).

### Cardiovascular gene therapies

The growing understanding of the molecular mechanisms regulating vascular and cardiomyocyte growth and homeostasis enables the development of targeted therapies for cardiovascular diseases. Gene therapy experiments in both small and large animals have provided promising results for enhancing cardiac vasculature and cardiomyocyte function. The most commonly used vectors for therapeutic gene delivery, adenoviral and adeno-associated viral vectors (AAVs) as well as plasmids, exhibit a good safety profile also in clinical trials. However, the clinical efficacy of cardiovascular gene therapies has been somewhat disappointing [reviewed in (62)]. Plasmids exhibit a rather low transfection efficiency, whereas adenoviral vectors have marked immunogenicity and induce high transient transgene expression. For ischemic heart disease, long-term transgene expression with low immunogenicity is needed, making recombinant AAVs currently the best choice. Although the AAV serotype AAV9 has the best tropism for cardiomyocytes (63), the lack of truly tissue-specific AAV vectors is a topical problem. Importantly for cardiovascular diseases, there are no rAAVs at the moment, which would transduce ECs efficiently.

Some randomized clinical trials with VEGF and FGF gene therapies have improved myocardial vascularity; however, convincing evidence on functional benefits is still missing [see (62, 64)]. Clinical trials targeting heart failure to enhance cardiomyocyte function have mainly used gene delivery of either SERCA2a or SDF-1, but the results have not fulfilled the expectations, possibly due to insufficient gene delivery (62, 65). This will likely improve in the near future, as re-engineered vectors will increase transduction efficiency and specificity, and more validated targets advance to clinical stage.

### RNA therapeutics

RNA-based drugs include short interfering RNAs, antisense oligonucleotides, messenger RNAs and CRISPR gene-editing technology (66). The first members of RNA therapeutics have gained FDA approval or entered advanced phases of clinical trials; most of them are for various cancers or viral infections (66). Thus far only one RNA drug targeting cardiovascular diseases is in clinical trials: AZD-8601 is a chemically modified RNA encoding VEGF for the induction of therapeutic revascularization in the heart (67). Its phase 1 trial (NCT02935712) has been completed and phase 2 trial (NCT03370887) has started recruiting patients (www.clinicaltrials.gov). It will be administered by epicardial injections during coronary artery bypass grafting surgery. In preclinical studies, several miRNAs have been shown to regulate EC and CM survival and proliferation and are thus expected to become potential therapeutic targets as the delivery and targeting techniques improve (68, 69).

### Heart repair by cell therapies and tissue engineering

Stem cell therapy for cardiac repair has been under intensive investigation and produces modest, although variable, improvements in cardiac function in clinical trials (70–73). The transplanted cells do not, however, differentiate into cardiac cells and engraft into the myocardium, but instead their effects are attributable to paracrine pro-survival factors. Transplantation of cardiomyocytes alone or together with other cell types has been studied in various preclinical models (74–77) including non-human primates (78, 79), and the results show engraftment of transplanted CMs into the myocardium and in most cases also improved cardiac function. The observed ventricular arrhythmias (78) call for caution before proceeding to clinical trials. In the majority of cell therapy experiments, the cells have been administered as injections and exhibit no organization at the time of transplantation, which may predispose to arrhythmias. A more refined approach, tissue-engineering, which produces cell sheets or patches with a more organized structure, i.e., cardiomyocyte alignment and established cell-cell connections, is proceeding to clinical trials in the near future [reviewed in (80)].

The requirement for fast neovascularization of the infarcted area (28) and experimental evidence showing that ECs improve the survival and spatial organization of CMs *in vitro* (81) suggest that concomitant transplantation of ECs together with CMs could improve the survival of transplanted cells and revascularization of the graft. However, intramyocardial injections of CMs in combination with ECs and SMCs did not improve cardiac function in a porcine model of MI despite enhancing vasculogenesis in the peri-infarct region (77). There is some evidence of improved outcome when ECs have been included in tissue-engineered patches (82), and patches containing CMs, ECs, and SMCs were recently shown to reduce infarct size and improve cardiac function in swine (83). However, ECs might be dispensable, as transplantation of patches consisting of CMs and fibroblasts also produces highly organized and progressively vascularized grafts in rat hearts (84).

Furthermore, the beneficial paracrine effects of cell therapy have prompted research on extracellular vesicles that carry pro-survival signals such as non-coding RNAs, which could be used to achieve similar effects as cell therapy without the delivery of cells. Prolonged cell-free delivery of hPSC-CM-derived extracellular vesicles into infarcted rat hearts promotes cardiac recovery (85). Furthermore, exosomes collected from cocultures of hPSC-derived CMs, ECs, and SMCs were reported to be more efficient in protecting cultured cardiomyocytes than exosomes from hPSC-CMs cultured alone (83) highlighting the importance of non-myocyte-derived signals in CM survival.

### Drug discovery

The efforts to discover regeneration-inducing pharmacological agents are still in their infancy. Considering the lack of regenerative gene expression response in both CMs and ECs (31), a potential regenerative treatment strategy would induce production of new CMs and promote revascularization at the same time. It is therefore very likely that a combination of compounds will be required. These may include compounds promoting cardiomyocyte proliferation, such as GSK3 inhibitors and p38 MAPK inhibitors (86) as well as proangiogenic factors or small molecule compounds, such as VEGFs or relaxin receptor agonists (87). Furthermore, compounds targeting the cardiac transcription factor GATA4, which promotes cardiac regeneration (88), might exhibit regenerative potential (89, 90).

Due to potential adverse effects in off-target organs, regeneration-inducing therapeutics should be specifically targeted to the heart. To avoid invasive administration, cardiac targeting with heart-homing nanoformulations has provoked interest. For example, porous silicon nanoparticles coated with ANP and loaded with a novel GATA4-targeted cardioprotective compound were shown to enrich in the ischaemic endocardium and inhibit prohypertrophic signaling after systemic administration (91). Although the current targeting efficiency requires improvement, nanoparticle-based drug delivery would not only enable tissue or cell type-specific targeting, but also prolonged or sequential release of several drugs, as well as enable the delivery of poorly soluble or instable therapeutics (92–94).

Cardiac heterocellularity also needs to be implemented in *in vitro* models used in drug discovery and development. More relevant *in vitro* systems are needed not only for screening and lead optimization of cardiovascular drugs, but also for screening cardiovascular and other drug candidates for cardiotoxicity and proarrhythmic effects. The advances in the production of human pluripotent stem cell-derived cardiovascular cells and microfabrication provide an unprecedented possibility for large-scale drug screening in humanized multicellular cardiac cell culture models and heart-on-a-chip systems (95, 96).

## Summary and concluding remarks

Cardiac regeneration requires an orchestrated multicellular response, the mechanisms of which are not yet fully understood (97). The current focus in the field of cardiac repair and regeneration has shifted from stem cells toward promoting regenerative processes in the endogenous cardiac cells, of which CMs and ECs are the most important considering regeneration. Detailed understanding of the mechanisms and crosstalk between these two cell types will be key in developing regenerative therapies.

## Author contributions

All authors listed have made a substantial, direct and intellectual contribution to the work, and approved it for publication.

### Conflict of interest statement

The authors declare that the research was conducted in the absence of any commercial or financial relationships that could be construed as a potential conflict of interest. The reviewer EA and handling Editor declared their shared affiliation.
